# Effects of swimming in cold water on lipolysis indicators via fibroblast growth factor-21 in male Wistar rats

**DOI:** 10.1016/j.bbrep.2024.101662

**Published:** 2024-02-13

**Authors:** Sara Shams, Mostafa Tavasolian, Sadegh Amani-Shalamzari, Pezhman Motamedi, Hamid Rajabi, Katja Weiss, Beat Knechtle

**Affiliations:** a Department of Exercise Physiology, Faculty of Sports Science, Kharazmi University, Tehran, Iran; b Medbase St. Gallen Am Vadianplatz, St. Gallen, Switzerland; c Institute of Primary Care, University of Zurich, Zurich, Switzerland

**Keywords:** Brown adipose tissue, Exercise, Cold stress, Weight loss, Temperature

## Abstract

This study aimed to investigate the effects of swimming in cold water on the release of FGF21 from various tissues and its impact on fat metabolism. Twenty Wistar rats were randomly divided into three groups: untrained (C), trained in thermo-neutral water (TN, 30 °C) and trained in cold water (TC, 15 °C). The training groups swam intervals (2–3 min) until exhaustion, 1 min rest, three days a week for six weeks, with 3–6% bodyweight load. The mRNA expression of variables was determined in white fat tissue (WAT), and FGF21 protein was also measured in the liver, brown fat tissue (BAT), serum, and muscle. The experimental protocols resulted in lower body weight gain, associated with reduced WAT volume; the most remarkable improvement was observed in the TC group. Swimming significantly increased FGF21 protein levels in WAT, BAT, and muscle tissues compared to the C group; substantial increases were in the TC group. Changes in FGF21 were highly correlated with the activation of genes involved in fat metabolisms, such as CPT1, CD36, and HSL, and with glycerol in WAT. The findings indicate a positive correlation between swimming in cold water and the activation of genes involved in fat metabolism, possibly through FGF21 production, which was highly correlated with fat-burning genes.

## Highlights

• Swimming in cold water intensifies the secretion of FGF21 from muscle, WAT, and BAT.
• Swimming in cold water activates genes involved in fat metabolism in WAT.
• Swimming in cold water activates the brown fat cells and help to manage the body weight.

## 1 Introduction

Extreme environments disrupt the body's homeostasis, leading to increased sympathetic nervous system activation [1]. Exposure to cold ambient stimulates the release of cortisol and norepinephrine (NE) [2], increasing the basal metabolic rate and enhancing mobility and oxidation of glucose and free fatty acids [3]. Repeated exposure to a cold environment may result in adaptive changes helping organisms to resist stress-induced damage [4]. Cold-stimulated cytokines such as fibroblast growth factor 21 (FGF21) may mediate these changes [5]. A 12-h exposure to a mild to cold environment (19 °C) compared to a moderate ambient (24 °C) environment led to increased plasma FGF21 levels in healthy adults. FGF21 was correlated with increased energy expenditures and lipolysis [6]. Therefore, the degree of cold exposure would determine the magnitude of responses and acclimatization [7].

FGF21 is a polypeptide involved in energy balance, glucose uptake, and lipid metabolism [8]. The predominant source of serum FGF21 is the liver, but it is released from white adipose tissue (WAT), brown adipose tissue (BAT) [9], and skeletal muscles [10]; however, it was reported that the predominant source of serum FGF21 is BAT in the cold environment [11]. FGF21 functions in an autocrine, paracrine, and endocrine manner by binding to its co-receptor βKlotho (KLB) [12] and causing a tissue cross-talk. It enhances glucose uptake by inducing glucose transporter 1 (GLUT1) expression via activating ERK1/2 in adipocytes [13,14] and myocytes [15]. Thus, it is involved in lowering insulin-independent blood glucose [15], improving lipid profile (increasing HDL and decreasing LDL) [16], and increasing adiponectin and bone formations markers [17]; so FGF21 may be effective for the treatment of metabolic disorders such as obesity and diabetes. In addition, it was shown FGF21 stimulates the expression of genes involved in lipolysis by increasing hormone-sensitive lipase (HSL) [18]. In this regard, increased lipolysis and lipogenesis in the Siberian hamster in response to treatment with FGF21 were reported [14]. On the other hand, it has been reported a short-term FGF21 treatment resulted in a marked increase in AMP-activated protein kinase (AMPK) and Peroxisome proliferator-activated receptor (PPAR)δ/γ signaling pathways, which in turn stimulates the expression of carnitine palmitoyltransferase I (CPT1), fatty acid translocase (CD36) [19] in BAT; hence it increases fat oxidation. However, less is known about the metabolic roles of FGF21 in cold exposure.

Acute and chronic exercise also affects the release of FGF21 from various tissues [12,20]. Research has shown that acute endurance exercise increases serum FGF21 levels in mice and healthy men [10,20]; in contrast, it was reported that the FGF21 protein content of the systemic circulation and skeletal muscle was unchanged in response to eccentric exercise [21]. Findings for regular exercise were contradictory and depended on the type of exercise, the duration of intervention, and exercise intensity. In this regard, an animal study showed an eight-week moderate-intensity training compared to high-intensity training was more effective at enhancing FGF21 and β-Klotho (KLB) expression in the liver, BAT, and muscle at both mRNA and protein levels [12]. In contrast, some studies reported that endurance training led to no changes or decreased serum and muscle FGF21 levels [22,23]. In rodents, it was reported that a period of moderate-intensity treadmill running did not significantly change serum FGF21 levels [24,25]. However, FGF receptor and co-receptor KLB expression were upregulated by exercise training in WAT and BAT [24,25] in obese mice. Therefore, there are conflicting findings regarding the effect of exercise training on FGF21 release.

Swimming in cold water is a stressful physiological condition that could exacerbate the body's response to exercise [26]; repeated cold-water swimming may result in beneficial adaptive changes in organisms' [26]. Increased metabolism and a thermogenic effect characterize exercise in a cold environment [26]. However, the potential effects of long-term swimming in cold water on physiological adaptations have not been studied, and most analyses used mild-cold water [27]. It was reported that a 5-week period of swimming in 24 °C water led to increased expression of mitochondrial biogenesis-related genes in soleus muscle and inguinal WAT of mice [28]. In addition, da Silva et al. (2020) showed eight weeks of swimming in mild-cold water (20 °C) does not exacerbate the independent effects of mild-cold exposure and swimming on browning-related markers in WAT and BAT in mice [27].

Therefore, it might be assumed that repeated cold-water swimming may amplify the potential effects of cold exposure and swimming on FGF21-stimulated metabolic indices. To verify this hypothesis, we examined the effects of swimming in cold water on FGF21 from secretory tissues, BAT, WAT, the liver and active muscle, and FGF21-stimulated fat metabolisms factors like AMPK, CPT1, CD36, and HSL in white fat tissue to determine whether there is a correlation between FGF21-primarily secreted tissues (the liver, muscle) with WAT. Thus this strategy may be used to manage overweight.

## 2 Materials and methods

### 2.1 Animals

Twenty male Wistar rats were housed in conventional cages and kept on a 12-h light/dark cycle in temperature-controlled situations. After two weeks of orientation, they were randomly divided into three groups: untrained (C, n = 6), trained in thermo-neutral water (TN, n = 7), and trained in cold water (TC, n = 7). The C group, as the control group, was sedentary during the intervention. The ambient temperature of the laboratory was 25 ± 2 °C. The TN group was kept at normal room temperature (25 ± 2 °C) and swam in the water with temperature (30 ± 2 °C), three days per week. The TC group was also kept at normal room temperature and swimming in cold water for three days per week. Using a few pieces of ice, the water was cooled to 15 ± 1 °C for the TC group. A waterproof digital thermometer was embedded in the water to display the target temperature. Animals were allowed ad libitum access to water and standard commercial chow. The same amount of chow for rats was put into the cages (50 g per rat) every time. Body weights were measured weekly by digital scale.

### 2.2 Incremental test

At the end of the protocol, an incremental swimming test was adapted from Almeida et al.'s study [29]. The test consisted of 3-min swimming intervals with increased loads separated by 1-min rests. Swimming started with an initial external load of 1, 2, and 3% of the rat's body weight, which fastened to the rat's tail for the first three stages, respectively. Then increments of 0.5% body weight in the following stages until animal exhaustion. Exhaustion was determined by the frequent submergence of rats. The percentage of body weight (%BW) that the rats could bear while swimming was used for statistical analysis.

### 2.3 Exercise protocol

The swimming protocol was conducted in a glass aquarium with a length of 100 cm, a width of 50 cm, and a water depth of 50 cm. The training protocol consisted of 2-min swimming intervals until exhaustion separated by 1 min of rest. The initial load was 3% of the rat's body weight, and it increased by 1% if they could swim ten successful repetitions. In addition, if they reached ten repetitions of 6% of their body weight, the work interval durations were increased to 3 min. They trained three times per week for six weeks [30]. The TN group could only swim ten repetitions with 3–6% of their body weights and swam 3-min intervals in the two last weeks. The TC group swam only 3% of their body weight, and the number of intervals increased from four to eight.

### 2.4 Blood and tissue sampling

The animals were anesthetized 48 h after the last session with ketamine (100 mg/kg) and xylazine (5 mg/kg). The rats were deprived of food 8 h before they were sacrificed, but they were allowed ad libitum access to water. Then, blood was immediately withdrawn intracardially. Blood samples were centrifuged for 10 min at 4000 rpm, and serum was gathered and stored at −20 °C. Subcutaneous white fat, interscapular brown fat, the liver, and soleus muscle were excised, and a slice of fats was fixed in 10% formaldehyde, passaged, and embedded in paraffin. A portion was frozen in nitrogen and stored at −80 °C.

### 2.5 Histological analysis

The white fat tissue was excised and fixed in 4% paraformaldehyde. The tissue samples were then dehydrated by the gradient concentrations of alcohol, cleared with xylene solvent, and embedded in paraffin. Paraffin blocks were sectioned (5–10 μm) by microtome (Leica, Germany), mounted on slides, deparaffinized, and stained with hematoxylin and eosin based on instructions. The stained tissue samples were visualized under a light microscope (Nikon, Tokyo, Japan), and the provided images were analyzed using Image J software. The average diameter of fat cells was measured using a graduated lens and suitable measurement software, such as Dyno Capture or Image G (Fiji). To calculate the histomorphometric parameter, 6 figures (n = 6) were used to obtain the results for each group. Also, the number of each adipocyte was counted in a 1 mm^2^ of area in each tissue section.

### 2.6 Protein assay

Frozen samples (100 mg) were homogenized in a mixed buffer containing 50 mM Tris-HCI (pH 7.8), 2 mM potassium phosphate, 2 mM EDTA, 2 mM GTA, 10% glycerol, 1% Triton X-100, 1 mM dithiothreitol, 3 mM benzamidine, 1 mM sodium orthovanadate, 10 mM leupeptin, 5 mg/ml aprotinin, and 1 mM 4 benzene sulfonyl fluoride. The homogenates were centrifuged at 12,000×*g* for 20 min at 4 °C, and the supernatant was removed, then protein concentrations were determined using the Bio-Rad protein assay. The samples were stored (−80 °C) and used for ELISA analysis. FGF21 levels in BAT, WAT, the liver, muscle, and serum were measured in duplicate by using specific ELISA kits (ZB-11362C, Zell Bio, Germany) according to the manufacturer's instructions [Sensitivity <5.5 pg/mL]. In addition, blood glucose (cat number: 9897956), glycerol (ZB-GCL-96A, Zell Bio, Germany), and NE (ZB-11137C, Zell Bio, Germany) concentrations were measured by specific kits.

### 2.7 Gene expression

Total RNA was extracted from 100 mg of WAT with Trizol solution according to the instructions (Invitrogen). RNA purity and quantity were confirmed by spectrophotometry using a NanoDrop ND-1000 (VWR, Radnor, PA, USA). A Qiagen cDNA synthesis kit (cat: K1622) was used for cDNA synthesis. QRT-PCR using the SYBR Green dye (Amplicon, 4309155) was performed to determine the mRNA relative expression of GLUT1, HSL, AMPK, CPT1, CD36, and KLB. The thermal cycling program was as follows: 95 °C for 15 min followed by 40 cycles of 95 °C for 0.5 min, 60 °C for 1 min, and 72 °C for 0.5 min. GAPDH mRNA was used as a normalized gene. The sequence of PCR primers is presented in Table 1. The 2^-ΔΔCT^ formula determined the fold change expression.

**Table 1:** The sequence of gene primers.

Gene name	Forward sequence	Reverse sequence	Amplicon size	Tm of PCR product
GLUT1	GATGTATGTGGGGGAGGTG	AGGGGTGGGATGAAGATGA	171	88
KLB	AGTTTGAGATCGCCTGGTTTG	TTCACCACCCTGCTCTCCTT	148	87
CPT1	GCTGGAGGTGGCTTTGGT	GCTTGGCGGATGTGGTTC	158	87
CD36	AACAACAAGGCCAGGTATCACAG	AGCTAGGCAGCATGGAACTTGAC	150	77
HSL	TCA CTG GTT TCA GCC TCT TCC	ATG AGA CAG CCC CGA GAT	135	80
GAPDH	AGGTCGGTGTGAACGGATTTG	TGTAGACCATGTAGTTGAGGTCA	123	83

### 2.8 Statistical analysis

The descriptive data are presented by mean ± standard deviation (SD). We used a one-way analysis of variance (ANOVA) to analyze the main effects of interventions on the variables. If significant results were obtained, Tukey post-hoc tests were done. To determine the magnitude and direction of the linear relationship between the serum marker and other variables, the bivariate Pearson correlation coefficient (r) was calculated. We considered the significance level at p ≤ 0.05 to accept the main effects. The Statistical Package of Social Sciences (SPSS, IBM, v19) was used to analyze the data.

## 3 Results

### 3.1 Bodyweight and fat tissue changes

**Fig. 1:**
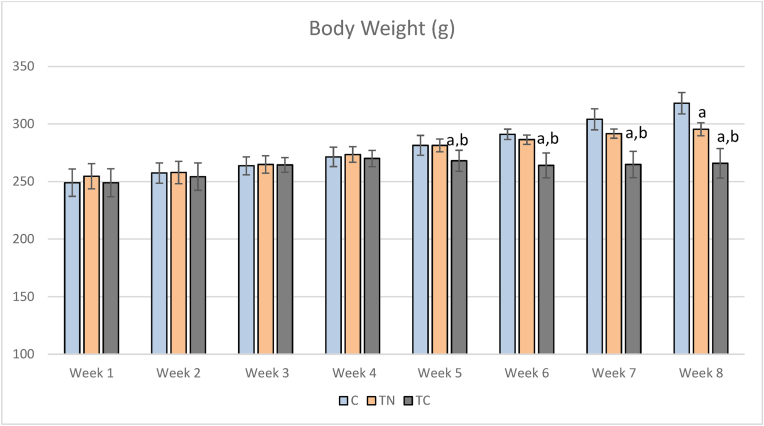
The rat's body weight during the intervention. C: Untrained, Trained in thermo-neutral water and TC: trained in cold water; a significant difference with the C group; b significant differences with the TN group.

At the start of the study, there were no differences in body weight between the groups (F = 0.38, p = 0.687). The body weight significantly changed during the research. Fig. 1 shows the trend of body weight changes during the orientation and an a-6-week period of intervention. Significant differences emerged from the fifth week until to end of the protocol (p < 0.001). In the fifth week (F = 4.75, P = 0.030), the significant difference was between the TC group with the two other groups. In the sixth week (F = 20.41, P = 0.001), and seventh week (F = 26.24, P = 0.001) the difference was between the TC group with the other groups (p < 0.001). The weight of the TC group decreased and remained constant, while the weight of the other two groups increased. In the last week (F = 36.45, P = 0.001), the significant difference was between the C group with the other trained groups (p > 0.001).

**Fig. 2:**
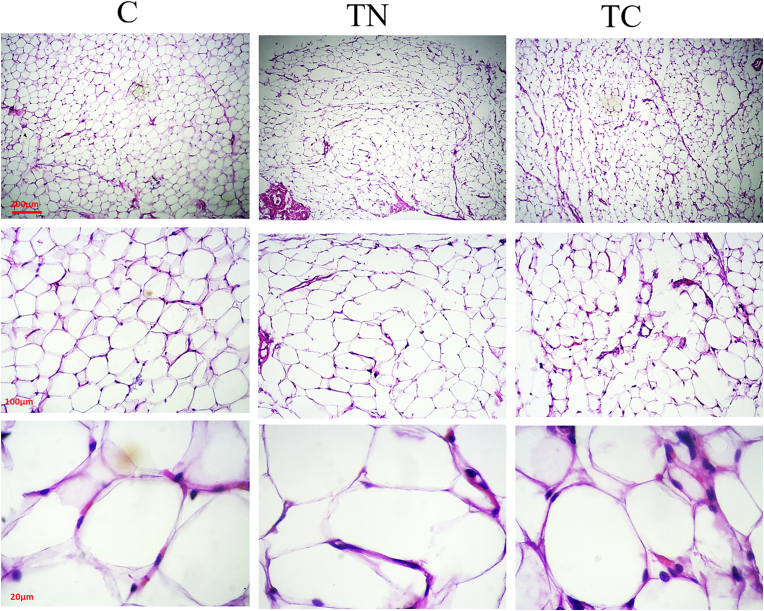
Subcutaneous white fat tissue in research groups. C: control, TN: Trained in thermo-neutral water, and TC: trained in cold water.

The changes in white adipose volume were in line with bodyweight changes. The diameter of fat white cells differed between groups (F = 39.43, *p* = 0.001, R^2^ = 0.83). As shown in Table 2, the largest diameter of white fat cells belonged to the C group (Fig. 2). The Tukey post-hoc test showed the volume of white fat cells in the C group was significantly different from the other groups (p < 0.05). There was no significant difference in the number of white fat cells between groups (F = 3.57, p = 0.061, R^2^ = 0.12). In addition, there was a significant correlation between body weight and the diameter of white fat cells (r = 0.64, p = 0.015).

**Table 2:** Serum glucose, norepinephrine, and physical features of rats.

	C	TN	TC
White fat diameter (μm/mm^2^)	51.53 (5.24)	37.35 (1.79)^a^	31.14 (3.30)^a^
White fat number (n/mm^2^)	12.20 (2.86)	9.40 (2.30)	13.20 (1.24)
Incremental test (%BW)	3.80 (0.27)	5.90 (0.55)^a^	5.20 (0.45)^a^
Glucose (mg/dl)	100.00 (8.18)	96.33 (4.16)	98.00 (7.21)
Norepinephrine (ng/ml)	2.32 (0.24)	2.76 (0.14)	2.89 (0.66)
Glycerol (ng/l)	61.72 (3.57)	133.26 (11.11)^a^	173.14 (14.33)^a,b^

C: Untrained, TN: Trained in thermo-neutral water and TC: trained in cold water; %BW: body weight percentage; a significant difference with C group; b significant differences with TN.

To determine the training efficiency, we used the incremental swimming test, and the weights that rats could bear while swimming are presented in Table 2. The duration of swimming to exhaustion in the C, TN, and TC groups were 13:00, 26:00, and 21:00 min, respectively. There was a significant difference between groups in the swimming to exhausting test (F = 29.82, p = 0.001, R^2^ = 0.92). The differences were between both training groups, TN and TC groups, with untrained group, the C group (*p* < 0.05).

### 3.2 Protein level

Table 2 presents the serum concentrations of NE, glucose, and white fat glycerol in groups. There was no significant differences between groups at NE levels (F = 2.53, *p* = 0.122, R^2^ = 0.29). There was also no significant difference between the groups in serum glucose levels (F = 0.14, *p* = 0.962, R^2^ = 0.04). The glycerol levels significantly differed between groups after the interventions (F = 139.90, *p* < 0.001, R^2^ = 0.96). The Tukey post-hoc test showed that the levels of glycerol in the two experimental groups had significantly increased compared to the C group (p < 0.01). Also, it was noticed that the TC group had a noticeable difference in glycerol levels compared to the TN group (p < 0.01). (Table 2).

**Fig. 3:**
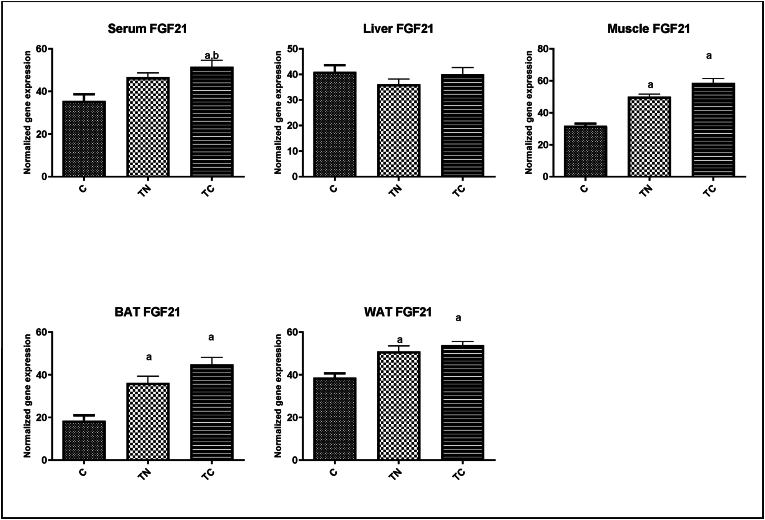
Level of FGF21 in the selected tissues in the groups. C: Untrained, TN: Trained in thermo-neutral water and TC: trained in cold water; WAT: white adipose tissue; BAT: brown adipose tissue. a significant difference with the C group; b significant differences with the TN group.

Fig. 3 presents the FGF21 protein levels in the selected tissues. Outputs of one-way ANOVA revealed there were significant differences between groups at FGF21 levels in some tissues. In soleus muscle (F = 41.47, *p* = 0.001, R^2^ = 0.93), the FGF21 protein levels in both trained groups, the TN (*p* = 0.002) and TC (*p* = 0.001), had significantly increased compared to the C group. In serum (F = 8.48, *p* = 0.018, R^2^ = 0.74), there was a significant increase in the TC group compared to the other two groups (*p* < 0.05). In WAT (F = 14.04, *p* = 0.005, R^2^ = 0.82), a significant difference was between the two trained groups, TC (*p* = 0.006) and TN (*p* = 0.016) groups, with the C group. In BAT (F = 19.24, *p* = 0.003, R^2^ = 0.86), the FGF21 protein levels in two trained groups, the TN (*p* = 0.015) and the TC (*p* = 0.002) groups, increased significantly compared to the control. However, there was no significant difference in the FGF21 level in the liver between the groups (F = 1.09, *p* = 0.395, R^2^ = 0.27).

**Fig. 4:**
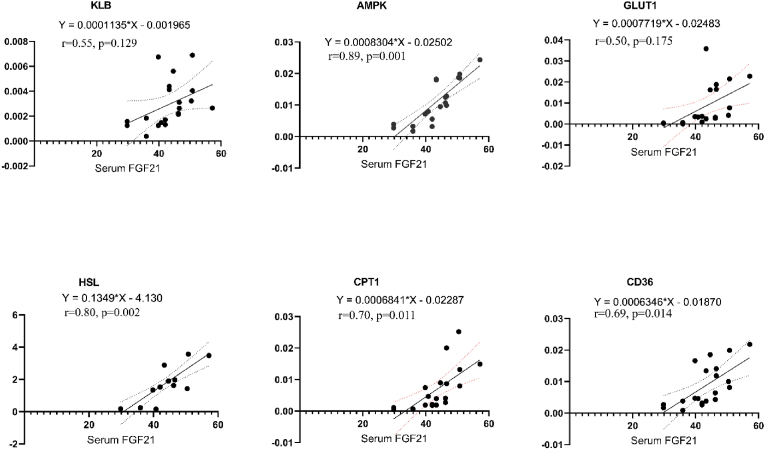
Correlation of serum fibroblast growth factor-21 (FGF21) levels with fat metabolism-involved gene expression in the white fat tissue. KLB: βKlotho; AMPK: AMP-activated protein kinase; GLUT1: Glucose transporter 1; HSL: Hormone-sensitive lipase; CPT1: Carnitine palmitoyltransferase I; CD36: Fatty acid translocase-36.

Serum FGF21 level was demonstrated to have significant positive correlations with FGF21 levels in BAT (r = 0.75, p = 0.005), WAT (r = 0.74, p = 0.006), and muscle (r = 0.77, p = 0.003), but not significant with the liver (r = 0.16, p = 0.618). Also, there was a large negative correlation between adipocyte size and concentrations of FGF21 in BAT (r = −0.79, p = 0.002), WAT (r = −0.73, p = 0.007), muscle (r = −0.73, p = 0.008), and serum (r = −0.66, p = 0.019), but not significantly with the liver (r = 0.25, p = 0.424). Fig. 4 shows the correlation between serum FGF21 levels with the genes involved in fat metabolism in WAT. There was a significant correlation between serum FGF21 with gene expression of AMPK (r = 0.89, p = 0.001); CD36 (r = 0.69, p = 0.014), and CPT1 (r = 0.70, p = 0.011) and HSL (r = 0.80, p = 0.002). However, there were no significant correlation between serum FGF21 with gene expression of KLB (r = 0.55, p = 0.129) and GLUT1 (r = 0.50, p = 0.175). In addition, there was a significant relation between serum FGF21 and glycerol levels in the WAT (r = 0.82, p = 0.001).

### 3.3 Gene expression

**Fig. 5:**
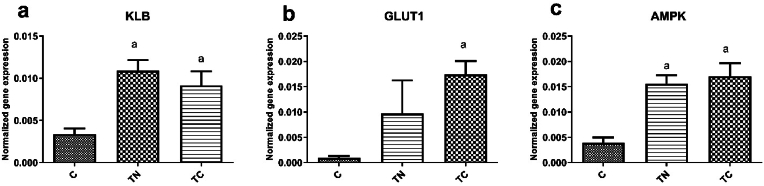
Gene expression of KLB (a), GLUT1 (b), and AMPK (c) in the white fat tissues. C: Untrained, TN: Trained in thermo-neutral water and TC: trained in cold water; WAT: white adipose tissue; KLB: beta-klotho, GLUT1: Glucose transporter 1; AMPK: AMP-activated protein kinase; a significant difference with the C group; b significant difference with the TN group.

As shown in Fig. 5 a, there was a significant difference in the KLB mRNA levels in WAT (F = 9.93, *p* = 0.001, R^2^ = 0.65). The Tukey post hoc test showed substantial differences between the C group with the TN group (*p* = 0.003) and with the TC group (*p* = 0.019). The KLB mRNA content were significantly increased in two training groups. Fig. 5 b shows the GLUT1 mRNA levels in WAT. Outputs of one-way ANOVA revealed significant differences in GLUT1 gene expression in the WAT (F = 4.07, *p* = 0.044 R^2^ = 0.40). Post-hoc analyses showed an upregulation of the GLUT1 gene in the TC group, which differed significantly from the C group in the WAT (*p=*0.037). The AMPK mRNA levels in WAT are also presented in Fig. 5 c. We found significant differences between groups in the AMPK mRNA levels in WAT (F = 14.75, *p* = 0.001, R^2^ = 0.71). The post-hoc test showed that AMPK mRNA expression increased significantly in the TC (*p* = 0.001) and TN (*p* = 0.002) groups compared to the C group.

**Fig. 6:**
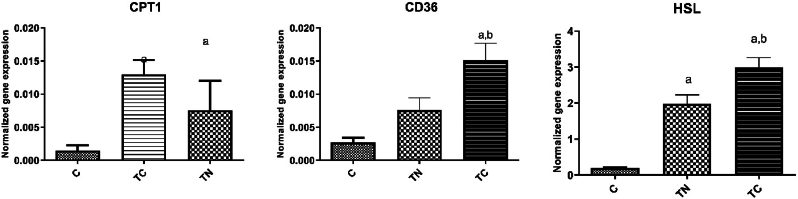
Gene expression of CPT1 (a), CD36 (b), and HSL (c) in the white fat tissues in the groups. C: Untrained, TN: Trained in thermo-neutral water and TC: trained in cold water; WAT: white adipose tissue; CPT1: carnitine palmitoyltransferase; CD36: fatty acid translocase; HSL: hormone-sensitive lipase. a significant difference with the C group; b significant differences with the TN.

Fig. 6 presents the mRNA level of genes involved in fat metabolism in WAT. There was a significant difference between groups in the CPT1 mRNA levels (F = 3.98, *p* = 0.048, R^2^ = 0.40), as shown in Fig. 6 a. The post-hoc test showed CPT1 mRNA expression increased significantly in the TC groups compared to the C group (*p* = 0.039). There were significant differences in CD36 mRNA levels (F = 11.55, *p* = 0.002, R^2^ = 0.66) between groups. The post-hoc test demonstrated that the increase rate in the TC group differed significantly from the C group (*p* = 0.001) and the TN group *p* = 0.033) in the WAT. Expression of HSL mRNA markedly differed between groups (F = 41.76, *p* = 0.001, R^2^ = 0.87), as shown in Fig. 6 c. The post-hoc test demonstrated that the upregulation of HSL expression in the TC group had differed significantly from the C group (*p* = 0.001) and the TN group (*p* = 0.018); in addition there is significant difference between the TN group with the C group (*p* = 0.001).

## 4 Discussion

This study hypothesized that the six weeks of cold-water swimming might magnify the potential effects of cold exposure and exercise on FGF21 production from various tissues and fat metabolism factors. The finding demonstrated that swimming resulted in lower body weight gain and reduced white adipose volume; swimming in cold water amplified these effects. Swimming in tepid water significantly increased FGF21 protein levels in WAT, BAT, and muscle tissue compared to the C group. In all tissues, TC group had relatively higher increase and only serum FGF21 levels had a significant difference between TC and TN groups. High correlations were observed between serum FGF21 and increased expression of KLB, AMPK, GLUT1, HSL, CPT1, and CD36 genes in the WAT. In addition, a positive impact of swimming in cold water on the upregulation of genes involved in fat metabolism was observed. Therefore, our finding partially confirmed the primary hypothesis that swimming in cold water might intensify the secretion of FGF21 from various tissues, and activates genes involved in fat metabolism in WAT. It may need more duration (>6 weeks) for meaningful effect.

Swimming in tepid water significantly increased the FGF21 levels in BAT, WAT, and muscle tissues compared to the C group. Hence, it seems that exercise training is an important factor in activating FGF21 in muscles and adipose tissues. In this regard, Xiong et al. (2020) showed that eight weeks of endurance training increased the expression of FGF21 from BAT, not WAT, in obese mice [12]. The effects of exercise on BAT activation are still unclear; however, some studies have proposed exercise training increases FGF21 secretion by repeated activation of the sympathetic system and NE secretion, activating BAT via the FGF21/PGC1α/UCP1 pathway [31,32]. In our study, we showed for the first time that swimming in tepid water led to an increase in the FGF21 protein in BAT and WAT and also KLB expression in the WAT. In this regard, Xiong et al. (2020) also reported increased KLB expression in adipose tissues and muscles of obese mice due to endurance training. In addition, a significant increase in the expression of AMPK and HSL genes in WAT following swimming in tepid water indicates swimming might primarily stimulate lipolysis in WAT through the FGF21/KLB signaling pathway. In contrast, the lack of a significant upregulation in CPT1 and CD36 genes in WAT probably indicates that oxidation lipid may need a longer training duration or cold stress in the WAT; however, adipose tissue is inactive during exercise training.

Compared to tepid water, swimming in cold water with less training duration caused a significant increase in FGF21 protein levels in the muscle, WAT, BAT, and serum, not the liver, compared to the C group. The increases in circulation were also significantly different from the TN group. When swimming in cold water, the body must overcome the cold stress in addition to the exercise load. With exposed to cold stress, stimulation of FGF21 secretion occurs through the NE-cAMP-dependent mechanism [33]. Exposure to a cold stress is a stressful situation, which releases stress hormones such as catecholamines [34] by activating the sympathetic nervous system, and leads to increase metabolism and non-shivering thermogenesis. Thus, swimming in cold water is a potent stimulus, through the release of NE, for the production and release of FGF21 into the bloodstream. The source of serum FGF21 concentration in the TC group could be considered as a considerable increase of FGF21 in muscle, BAT, and WAT. However, a decrease [23] or no change [35] and an increase [36] in serum FGF21 concentration were reported following prolonged training. Therefore, there is a positive correlation between swimming in cold water and increasing FGF21 from tissues and its release into the circulation. Although the liver is the primary source of FGF21 release, the effect of regular exercise on hepatic FGF21 expression is unclear. Research has reported increased hepatic FGF21 expression immediately after an acute exercise [20], but the chronic effects of training on hepatic FGF21 expression have not been studied. In this study, regular swimming had no impact on hepatic FGF21 levels. Therefore, the lack of significant correlation between serum FGF21 and the liver may be that the release of FGF21 from the liver did not respond to interventions and the liver produces a constant amount of FGF21. In addition, swimming in cold water increased KLB gene expression in WAT compared to the C group. Studies have reported KLB gene expression up-regulates via activating PPRG in WAT [24,25] in obese mice following moderate to high-intensity endurance training, and KLB gene expression up-regulates in the liver, muscle, and BAT, but not WAT after moderate endurance training [12]. Therefore, the duration and intensity of exercise and cold stress affect the KLB gene expression from fat tissues.

The findings showed that GLUT1 expression was increased in the TC intervention compared to the C groups. However, swimming had a relative effect on GLUT1 expression in WAT, but swimming in cold water caused to significantly increase in the GLUT1 expression in fat tissue. Researchers have shown that three weeks of training was associated with increased GLUT1 expression in WAT [37]. However, in our study, swimming in cold, not tepid water, increased the expression of the GLUT1 gene in WAT. Therefore, due to the lack of research that explored the expression of GLUT1 following swimming in cold water in WAT, more studies are needed to provide a definitive result.

Moreover, swimming in cold water significantly enhances the expression of HSL, CPT1, and CD36 in WAT compared to the C group; hence, lipid metabolism might increase. As the metabolic rate increases almost threefold during exposure to cold water [38], and FGF21 is one of the stimulants of increasing metabolism and energy homeostasis [13,15,19] during exposure to cold stress, the upregulated HSL, CD36, and CPT1 may be attributed partly to the increased FGF21 [19,39]. This confirms our observations of the high correlation between FGF21 levels and these genes. High levels of glycerol in this group also confirm the occurrence of lipolysis. Therefore, the lower weight gain observed in the TC group could be attributed to the high fat-burning rate.

The findings showed that following the training protocol; there was a significant increase in swimming repetitions to exhaustion in the training groups compared to the untrained group. Regardless of water temperature, swimming training increases aerobic capacity, delays fatigue, and prolongs exercise duration; this finding is consistent with previous animal studies [27,29]. Moreover, rats swimming in lukewarm water (30 °C) improved swimming to exhaustion compared to cold water. Swimming in cold water is a stressful situation. Thus, substantial energy is spent on overcoming cold stress, such as higher oxygen demand, maintaining temperature, and increased metabolism [40]; therefore, fatigue occurs earlier. As a result, aerobic fitness's magnitude is not the same as training in natural water.

We acknowledge that there were some limitations in the present study. First of all, we did not have access to devices that measure the body composition of rats, which could be of great help in interpreting the data. Secondly, blood fatty acids were not measured in this study. The lack of measurement of our variables at the end of the first week was another limitation of this study because we observed acute weight loss of animals in the first week in the group exposed to cold ambient. Therefore, in a future study, it is suggested to measure the acute effect of such interventions on the lipolysis rate by measuring fatty acids and glycerol in the blood.

## 5 Conclusion

Overall, our findings showed that swimming in cold water had a favorable impact on managing body weight by burning fat tissues. In addition, the TC intervention causes an increase in the release of NE-induced FGF21 from various tissues, especially serum, which has a positive relationship with the indicators related to the lipolysis, transfer, and oxidation of fats glucose transfer in WAT. Although swimming in cold water may release many cytokines involved in fat metabolism, we highlighted the FGF21 signaling pathway as one of the possible pathways in this study. Therefore, swimming in cold water may increase the secretion of FGF21 from all tissues in a longer duration. Overall, due to the findings in the TC group with less swimming volume, interval swimming in cold water is recommended for weight loss.

## Funding

This research received no external funding

## Ethical approval

The Sport Sciences Research Institute of Iran (approval number: IR.SSRI.RE.1400.964) approved all research procedures. This study was conducted in accordance with the National Research Council's Guide for the Care and Use of Laboratory Animals.

## Informed consent

Not applicable.

## CRediT authorship contribution statement

**Sara Shams:** Conceptualization. **Mostafa Tavasolian:** Conceptualization. **Sadegh Amani-Shalamzari:** Conceptualization. **Pezhman Motamedi:** Conceptualization. **Hamid Rajabi:** Conceptualization. **Katja Weiss:** Writing – review & editing. **Beat Knechtle:** Writing – review & editing.

## Declaration of competing interest

The authors declare that they have no known competing financial interests or personal relationships that could have appeared to influence the work reported in this paper.

## Data Availability

Data will be made available on request.
